# Species-Specific Transcriptional Regulation of Genes Involved in Nitric Oxide Production and Arginine Metabolism in Macrophages

**DOI:** 10.4049/immunohorizons.1700073

**Published:** 2018-01-01

**Authors:** Rachel Young, Stephen J. Bush, Lucas Lefevre, Mary E. B. McCulloch, Zofia M. Lisowski, Charity Muriuki, Lindsey A. Waddell, Kristin A. Sauter, Clare Pridans, Emily L. Clark, David A. Hume

**Affiliations:** * The Roslin Institute and Royal (Dick) School of Veterinary Studies, University of Edinburgh, Easter Bush, Midlothian EH25 9RG, United Kingdom; † Mater Research–University of Queensland, Translational Research Institute, Woolloongabba, Brisbane, Queensland 4102, Australia

## Abstract

Activated mouse macrophages metabolize arginine via NO synthase (NOS2) to produce NO as an antimicrobial effector. Published gene expression datasets provide little support for the activation of this pathway in human macrophages. Generation of NO requires the coordinated regulation of multiple genes. We have generated RNA-sequencing data from bone marrow–derived macrophages from representative rodent (rat), monogastric (pig and horse), and ruminant (sheep, goat, cattle, and water buffalo) species, and analyzed the expression of genes involved in arginine metabolism in response to stimulation with LPS. In rats, as in mice, LPS strongly induced *Nos2*, the arginine transporter *Slc7a2*, arginase 1 (*Arg1*), GTP cyclohydrolase (*Gch1*), and argininosuccinate synthase (*Ass1*). None of these responses was conserved across species. Only cattle and water buffalo showed substantial *NOS2* induction. The species studied also differed in expression and regulation of arginase (*ARG2*, rather than *ARG1*), and amino acid transporters. Variation between species was associated with rapid promoter evolution. Differential induction of *NOS2* and *ARG2* between the ruminant species was associated with insertions of the Bov-A2 retrotransposon in the promoter region. Bov-A2 was shown to possess LPS-inducible enhancer activity in transfected RAW264.7 macrophages. Consistent with a function in innate immunity, NO production and arginine metabolism vary greatly between species and differences may contribute to pathogen host restriction.

## Introduction

The ability of rodent macrophages to produce NO through the metabolism of arginine was described in the late 1980s (1) and the cDNA encoding the calcium-dependent, inducible enzyme required for this activity, now known as NO synthase (NOS2), was isolated soon afterward (2, 3). Subsequently, the *Nos2* gene was deleted in the mouse germ line, and shown to be required for optimal host defense against mycobacteria (4) and for numerous other intracellular pathogens and pathogenic processes. A current search of PubMed for “NO AND macrophage” produces ~18,000 hits. Throughout that vast literature, the species being examined is commonly omitted from the title of the work. Yet, almost from the outset, it was clear that there are major species differences in macrophage arginine metabolism and the production of NO. In a recent review, Bogdan (5) stated that “there is no doubt that human cells are able to express NOS2 protein and activity in vitro and in vivo.” However, the data supporting human macrophage NOS2 protein expression in vivo rely heavily upon detection with commercial polyclonal antisera (e.g., Ref. 6). The large majority of published studies where there has been direct comparison with mouse have found little or no detectable *NOS2* mRNA or NO production in human monocytes or macrophages stimulated in vitro (e.g., Ref. 7). Gross et al. (8) found that the *NOS2* promoter region is methylated and contained in inactive chromatin in human alveolar macrophages. Inactive chromatin status at *NOS2* is also evident in freshly isolated human blood monocytes (9). In the large FANTOM5 dataset, based upon deep sequencing of CAGE libraries, *NOS2* mRNA was not detectable in human monocyte-derived macrophages stimulated with LPS, or in fresh monocytes stimulated with a wide range of stimuli. In fact, the most abundant site of expression was adipocytes (10). Vitek et al. (11) created a human *NOS2* transgene on a mouse *Nos2*-deficient background, and reported that both *NOS2* expression and inducible NO production in macrophages were considerably lower than in *Nos2*^+/+^ mice. Substantial differences in the set of LPS-inducible genes between humans and mice can be associated with major differences in promoter architecture; regulatory elements identified in mice are not conserved in humans (7). The regulatory differences between mouse and human macrophages are not restricted to *NOS2*, and are shared with other species. Pig macrophages also failed to induce *NOS2* mRNA in response to activation (12), but share with humans the induction of a substantial set of genes that are not induced in mouse. *Nos2* induction is not even uniform among rodent species. Isolated macrophages from guinea pigs and hamsters produce substantially less NO than mice, and this has been associated with *Nos2* mRNA and promoter variation (13–15). Other species in stimulated macrophages appear to produce little or no detectable *NOS2* activity include rabbits, sheep, goats, monkeys, horses, and badgers (15–18).

Mice, humans, and other species also differ in other aspects of arginine metabolism. The production of NO in mouse macrophages depends upon induction of the cationic amino acid transporter encoded by *Slc7a2* (19), which is not detectably expressed in human myeloid cells (7, 10), or in activated pig macrophages (12). Degradation of arginine by arginase enzymes potentially competes for intracellular arginine to compromise NO production. Thomas and Mattila (20) reviewed the literature on arginine metabolism in human macrophages. In mice, arginase 1 (*Arg1*) has come to be regarded as a marker for M2/IL-4–mediated macrophage polarization, but it is not shared with human macrophages (21). Indeed, in the FANTOM5 CAGE data, *ARG1* mRNA in humans is very strongly expressed by neutrophils, as well as hepatocytes and the liver, but is entirely absent from monocytes and macrophages in any state of activation (10). Finally, the key cofactor for NOS2, tetrahydrobiopterin (THB4), is regulated differently between the species. In both mouse and human, the limiting enzyme GTP cyclohydrolase 1 (GCH1) is strongly inducible in macrophages. However, in human monocytes the downstream enzyme, 6-pyruvoyl THB4 synthase (PTS), was expressed at very low levels, and the major outcome of *GCH1* induction was the production and secretion of neopterin (22, 23). In this study we take advantage of large RNA sequencing (RNA-seq) datasets from multiple species to reexamine the species specificity of genes involved in arginine metabolism, and analyze the promoters of differentially regulated transcripts to highlight possible mechanisms underlying the gain and loss of gene expression.

## Materials and Methods

### Animals

Approval was obtained from the Protocols and Ethics Committees of The Roslin Institute, The University of Edinburgh, and the Royal (Dick) School of Veterinary Medicine. In accordance with the United Kingdom Animal (Scientific Procedures) Act 1986, this study did not require a Home Office project license as no regulated procedures were carried out. Cattle, water buffalo, and pigs were euthanized by captive bolt, sheep were euthanized by electrocution and exsanguination, and rats were euthanized by CO_2_ asphyxiation. Goat samples were collected from the slaughter-house. Horses were admitted to the Equine Hospital at the Royal (Dick) School of Veterinary Studies for elective euthanasia. Horses were euthanized with i.v. secobarbital sodium 400 mg/ml and cinchocaine hydrochloride 25 mg/ml (Somulose; Arnolds/Dechra).

### Generation of bone marrow–derived macrophages

Ribs were collected postmortem from cattle, goats, horses, sheep, pigs, and water buffalo, and femurs were collected postmortem from rats. Bone marrow (BM) cells were isolated using the methods described by Schroder et al. (7) and Kapetanovic et al. (12). BM-derived macrophages (BMDM) were cultured from cryopreserved BM cells for each species. Briefly, BM cells isolated from rats were cultured in DMEM (Sigma-Aldrich), heat-inactivated 10% FBS (GE Healthcare), penicillin/streptomycin (Thermo Fisher Scientific), and GlutaMAX (Thermo Fisher Scientific). BM cells from pigs were cultured as described by Kapetanovic et al. (12), sheep cells were cultured as described by Bush et al. (24), and cells from all other animals were cultured in RPMI 1640 (Sigma-Aldrich), heat-inactivated 20% FBS (GE Healthcare) (cattle and water buffalo) or autologous serum (Sigma-Aldrich) (goat and horse), penicillin/streptomycin (Thermo Fisher Scientific), and GlutaMAX (Thermo Fisher Scientific). BMDM were obtained by culturing BM cells for 7–10 d in the presence of recombinant human CSF1 (10^4^ U/ml; a gift from Chiron, Emeryville, CA) on bacteriological plates, as described in mouse, pig, and sheep (7, 12, 24); goat BMDMs were differentiated on tissue culture plastic. Differentiated macrophages were detached from plates by either vigorous washing using a syringe and blunt 18 g needle, or using a cell scraper, then washed, counted, and reseeded at 10^6^ cells per ml. Cells were treated with LPS from *Salmonella enterica* serotype Minnesota Re 595 (Sigma-Aldrich) for 7 and 24 h at a final concentration of 100 ng/ml for large animals and 10 ng/ml for rats.

### RNA isolation

RNA was isolated from control and LPS-stimulated cells using the TRIzol method (Thermo Fisher Scientific) followed by a clean-up step from the RNeasy Mini Kit (Qiagen). Cells were lysed in six-well plates at 0, 7, and 24 h post-LPS stimulation with 1 ml TRIzol, then frozen until RNA extraction was performed. Tissue culture replicates were included. Lysates were thawed and brought to room temperature. Chloroform (200 μl) was added and samples incubated for 2–3 min at room temperature. The samples were centrifuged at 12,000 × *g* at 4°C for 15 min to separate the phases. The aqueous phase was collected then precipitated in 1 volume of 70% ethanol. Samples were then transferred immediately to an RNeasy Mini Kit spin column and clean-up performed as specified by the manufacturer. RNA was quantified by Qubit BR dsDNA assay (Thermo Fisher Scientific) and RNA integrity number equivalent was calculated using RNA ScreenTape on the Agilent 2200 TapeStation. All samples had RNA integrity number equivalent values >7.

### Library preparation and sequencing

RNA-seq libraries were generated and sequenced by Edinburgh Genomics. All libraries were prepared using the Illumina TruSeq Stranded library protocol for total RNA libraries (Part: 15031048, Revision E) with the exception of rat and goat where stranded mRNA libraries were prepared (Part: 15031047, Revision E). TruSeq Stranded total RNA libraries were sequenced at a depth of >100 million paired-end reads per sample for cattle, buffalo, horse, and pig using the Illumina HiSeq 2500 platform. Similarly, TruSeq Stranded mRNA libraries were sequenced at a depth of >25 million paired-end reads for rat on the Illumina HiSeq 2500 platform. The sheep RNA-seq dataset is a component of a high resolution atlas of gene expression for sheep, which we have described previously (25). Goat mRNA libraries were sequenced at a depth of >50 million paired-end reads per sample using the Illumina HiSeq 4000 platform. Raw read data for all libraries has been submitted to the European Nucleotide Archive (https://www.ebi.ac.uk/ena) under accession numbers PRJEB19199 (sheep), PRJEB21180 (water buffalo), PRJEB22535 (cattle), PRJEB22536 (pig), PRJEB22537 (horse), PRJEB22553 (rat), and PRJEB23196 (goat).

### RNA-seq data processing

RNA-seq data were processed using the high-speed transcript quantification tool Kallisto v0.43.0, as described previously (24), generating gene-level expression estimates as transcripts per million (TPM). Kallisto quantifies expression by building an index of k-mers from a set of reference transcripts and then mapping the reads to these directly (26). The reference transcriptomes for each species, from which Kallisto indices were generated, are given in Supplemental Table I. A two-pass approach to Kallisto was used (24) whereby these transcriptomic indices are iteratively revised and expression requantified. In brief, expression was quantified for an initial analysis (the first pass), the output of which is parsed so as to revise the transcriptome. A second index is then created with a higher proportion of unique k-mers, conferring greater accuracy when (re) quantifying expression. The revised indices include, where possible, de novo assembled transcripts that had not previously been annotated [by taking the set of reads Kallisto could not map during the first pass and assembling them with Trinity version r20140717 (27)] and exclude transcripts not detectably expressed in any library during the initial analysis (detailed in Supplemental Table I). For both the first and second pass index, k = 31. The expression of genes involved in arginine metabolism (KEGG pathway ID: map00230; http://www.genome.jp/kegg/pathway/map/map00330.html) was then compared across species.

### Griess assay

NO production was measured by Griess assay. Nitrite (the product of NO oxidation in culture) was quantified against sodium nitrite standards. Cell culture supernatants from LPS-treated BMDM were added to an equal volume of Griess reagent [1% sulfanilamide, 0.1% *N*-(1-naphtyl) ethylenediamine diHCl, 2.5% phosphoric acid]. The reaction was incubated at 37°C for 30 min then the absorbance measured at 570 nm. As a positive control for NO production, chicken BMDM—prepared as previously described (28)—were stimulated with LPS under the same conditions.

### Bovine NOS2 promoter (enhancer) assay

A 150 bp region of the bovine NOS2 promoter covering the Bov-A2 element was synthesized by Eurofins Genomics and cloned into the *BamH1/Sal1* site downstream of the promoter-luc+ transcriptional unit of the pGL3 promoter vector (E1761; Promega). Transient transfections were performed by electroporation of 5 × 10^6^ RAW 264.7 cells with 5 μg of pGL3-NOS2 construct or empty vector in 0.4 cm electroporation cuvettes at 300 V, 950 μF using a Bio-Rad Gene Pulser. Transfected cells were cultured at 37°C for 4 h then given fresh media and returned to the incubator overnight. The following day, cells were treated with 100 ng/ml LPS and incubated at 37°C. Control wells containing no LPS were incubated in parallel. After 24 h LPS stimulation, the media were removed, and the cells washed in PBS then lysed in Luciferase assay lysis reagent (E4030; Promega) at −80°C for 1 h. The cells were collected from the plates by scraping, then the lysates collected in microfuge tubes and vortexed for 10–15 s. The samples were centrifuged at 12,000 × *g* for 15 s at room temperature then the supernatants collected for luciferase assay. Luciferase reagent was dispensed into an opaque 96-well plate. Then 20 μl of each sample was added to the wells containing the reagent and the plate vortexed briefly. The plate was analyzed on a Synergy HT Biotek luminometer.

## Results

### Species differences in NO production

To extend our knowledge of the diversity and evolution of innate immune genes across species, we have adapted methods previously described for the mouse and pig (12) for the production of BMDM from rat, horse, sheep, goat, cattle, and water buffalo. For this study, pig BMDMs were also generated for RNA-seq analysis. In each case, BM cells were grown in recombinant human CSF1 for 7–10 d, after which the cells form a relatively confluent population of macrophages. The cells were then harvested from their culture dishes and counted before reseeding on tissue culture plastic for stimulation with LPS. For each species, we have determined the time course of activation by measuring the inducible expression of *TNF-α* mRNA.

**Figure 1:**
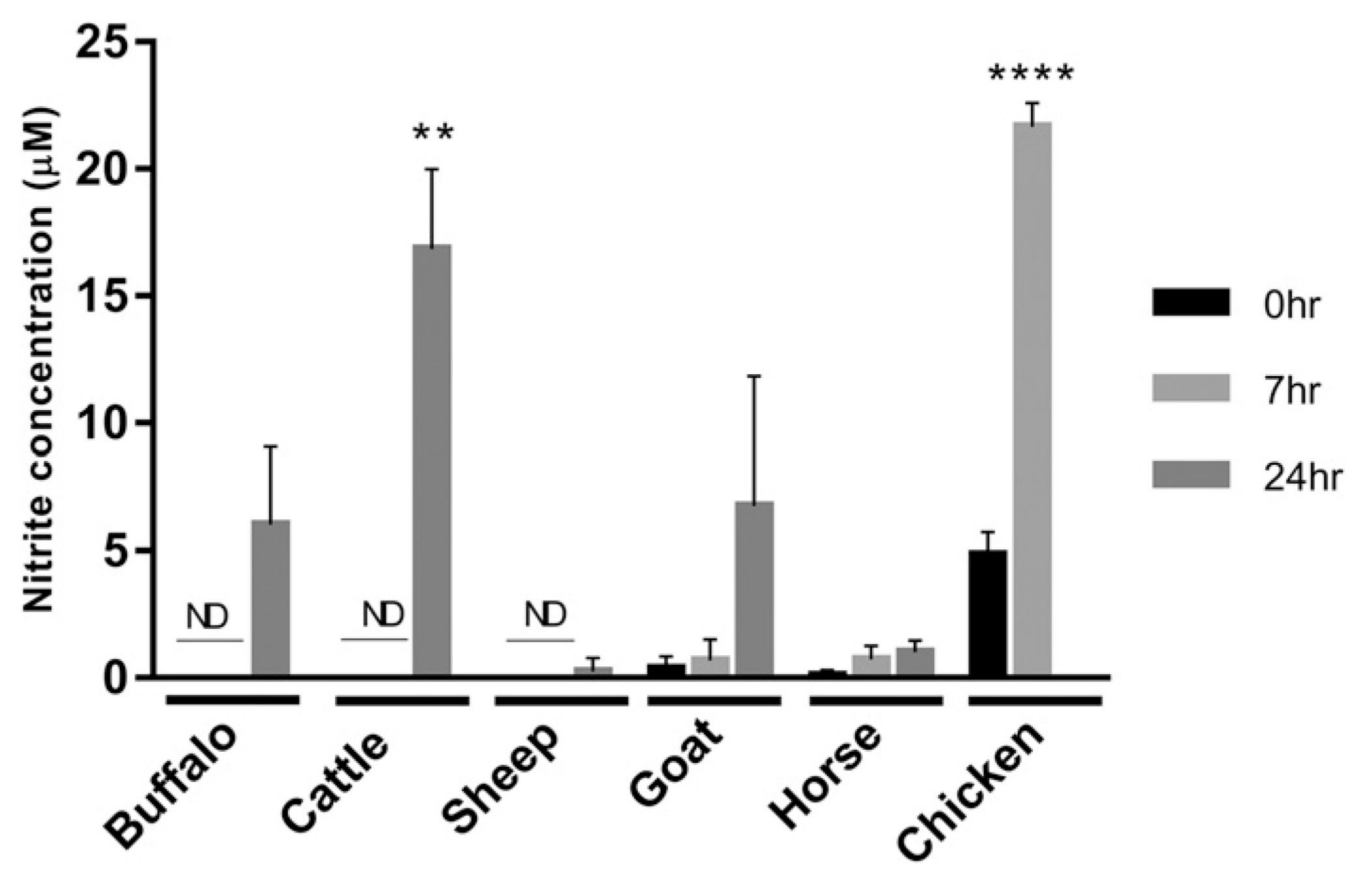
LPS-inducible NO production in macrophages. Supernatants were collected from LPS stimulated (*S. enterica*; 100 ng/ml) BMDM from water buffalo, cattle, sheep, goat, and horse at 0, 7, and 24 h poststimulation and nitrite production measured by Griess assay. Stimulated chicken BMDM 0 and 7 h poststimulation were used as a positive control. Mean nitrite levels are shown with error bars for the SD of the mean for three biological replicates per species, performed in duplicate. Statistically significant differences versus unstimulated cells are indicated (*t* test; ****p < 0.0001, **p < 0.01). ND, not detected.

To confirm and extend previous findings, we examined LPS-inducible NO production in a number of species. Fig. 1 shows comparative analysis of water buffalo, cattle, sheep, goat, and horse responses to LPS. In each case, a positive control, chicken BMDM, which we have previously shown produce large amounts of NO in response to LPS (28), was tested side by side. Cattle macrophages made NO in response to LPS treatment at levels similar to chicken BMDM; under similar conditions, water buffalo and goat made lower levels of NO, and sheep macrophages produced no detectable NO. Horse BMDM produced no detectable NO in response to LPS, as previously noted for alveolar macrophages (18). In mice, NO production can also be induced by IFN-γ, and this treatment increases the response to LPS, largely by shifting the LPS dose-response curve rather than increasing the absolute response (29). However, horse macrophages made very low levels of NO even after IFN-*γ* priming (Supplemental Fig. 1).

### RNA-seq analysis of genes involved in arginine metabolism

**Figure 2:**
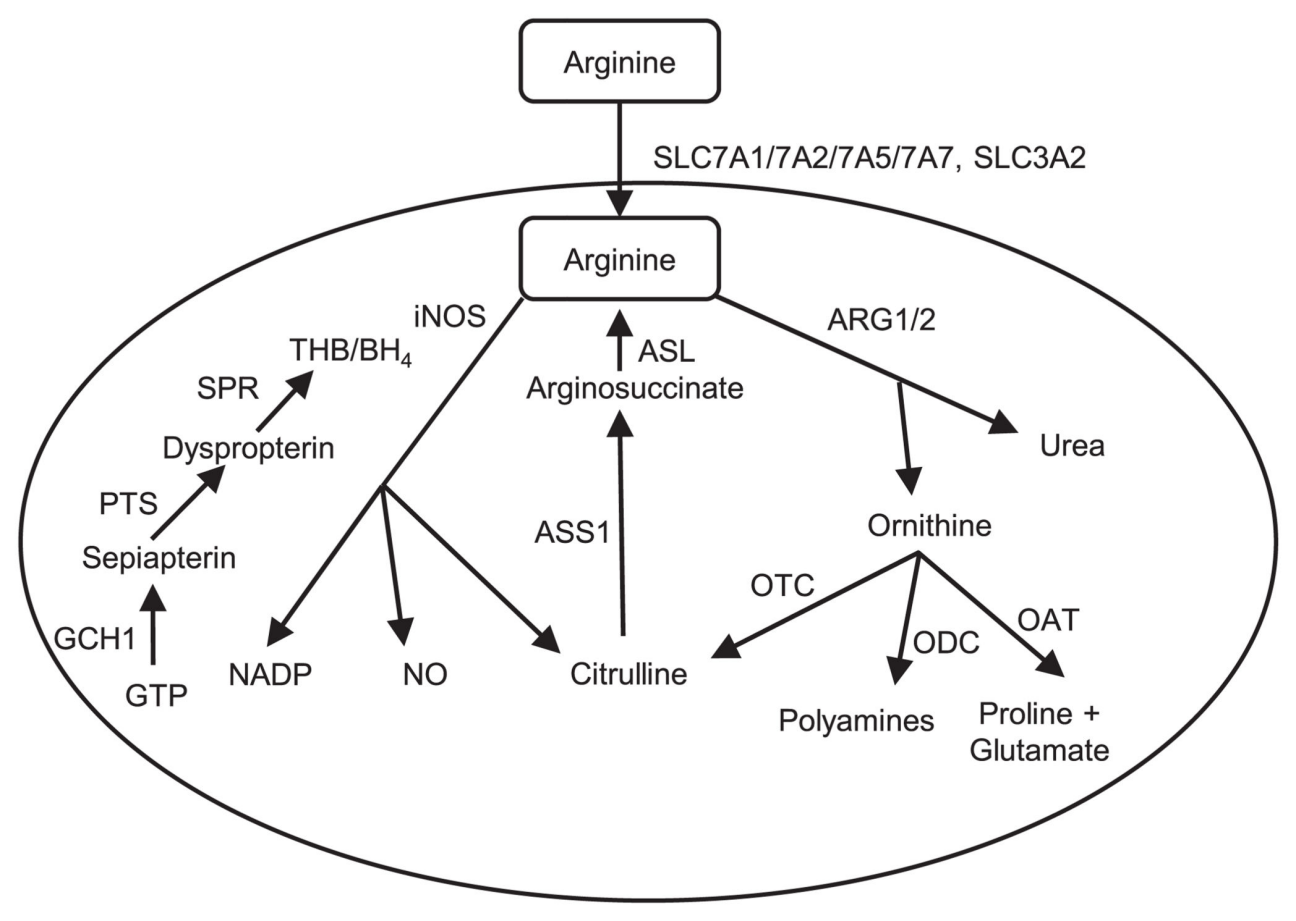
Mammalian arginine metabolism pathway in macrophages. Arginine is transported into mammalian macrophages by amino acid transporters (SLC7A1/7A2/7A5/7A7, SLC3A2), then metabolized by either inducible NO synthase (iNOS) into NO and citrulline, or arginase into ornithine and urea. Citrulline can feed back into arginine synthesis via ASS1 and inducible ASL. Citrulline, polyamines or proline and glutamate can be generated from ornithine via ornithine transcarbamylase (OTC), ornithine decarboxylase (ODC) or ornithine aminotransferase (OAT), respectively. The iNOS cofactor, THB, is generated by GTP via GCH1, PTS, and SPR respectively, and is a rate-limiting step in the production of NO.

There are several possible reasons why macrophages might not make detectable NO, even if *NOS2* mRNA is induced. We therefore examined the expression of all relevant genes in each of the species. For comparison across species, we chose the 7 h time point following LPS addition, consistent with a previous comparative analysis of mouse, pig, and human (12). In the current study, we included rat, rather than mouse, as a positive control rodent species, in part also to determine whether the mouse is representative as a rodent species (see Introduction). Fig. 2 summarizes the pathways of mammalian arginine metabolism, and Table I shows the expression levels of transcripts encoding enzymes associated with arginine metabolism and the production of the NOS2 cofactor, THB4. For comparison, we have extracted expression of these genes from the FANTOM5 CAGE tag sequencing dataset on human monocyte-derived macrophage response to LPS; the data are consistent with previously published microarray data (7).

There are a number of features to note. Firstly, all four of the ruminant species induced *NOS2* mRNA in response to LPS, but the maximum levels of stimulated expression were at least 15-fold lower in sheep and goats (TPM≈20) compared with water buffalo (TPM >300), and cattle produced even higher levels of mRNA (TPM≈900). The induced level of *NOS2* mRNA in sheep [see also BioGPS sheep dataset (www.biogps.org/dataset/BDS_00015/sheep-atlas/)] and goat macrophages was lower than the unstimulated level in rat macrophages (TPM≈60). It is unclear why goat macrophages produced detectable NO, where sheep macrophages did not. One explanation may lie in the relatively high expression of genes required for cofactor, THB4, production (*PTPS, SPR*) in goat macrophages. Horse and pig *NOS2* mRNA was on the limits of detection (TPM <2), although following LPS stimulation, rat *Nos2* mRNA was a further order of magnitude higher than in any of the ruminants (TPM≈5000). The second key difference between all of the large animals and rodents is the regulation of genes involved in arginine uptake. In rats, as in mice (19), LPS greatly increased (18-fold) expression of the cationic amino acid transporter, *Slc7a2*, whereas goats were the only large animal species in which *SLC7A2* mRNA was detectable (TPM 6) and regulated to any degree by LPS, with an induced level (TPM≈16) still lower than the basal level in the rat (TPM 46). Goat and buffalo also expressed the other cationic arginine transporter, *SLC7A1*, at higher level, inducible in buffalo and constitutive in goat.

The major avenue for arginine uptake in large animal macrophages is likely to be SLC3A2/SLC7A7 (also known as the Y+L or LAT1/CD98 system), which also mediates the uptake of other large neutral amino acids, including tryptophan, and is likely to be involved in inducible tryptophan metabolism. The gene encoding the alternative L chain, *SLC7A5*, was only expressed at high levels in the goat macrophages (TPM≈115). *SLC3A2* and *SLC7A7* were both highly expressed in macrophages from all of the large animal species examined, including humans, substantially higher than in rats. In humans at least, *SLC7A7* is strongly monocyte-macrophage enriched relative to other cell types and tissues (10); see also data on the BioGPS web portal (www.biogps.org/dataset/GSE1133).

The species studied also differ greatly in their expression of mRNA encoding genes involved in arginine breakdown. *Arg1* has been proposed as a mouse M2 macrophage marker and is strongly inducible by IL-4, but induction was not conserved in human macrophages (21). *Arg1* was induced by LPS in mouse macrophages, but not in human (7). In rat macrophages, *Arg1* was highly expressed and very strongly induced by LPS. In the FANTOM5 dataset (10) neither *ARG1* nor *ARG2* was expressed in human monocytes or macrophages in any activation state. In pigs, as in rats, *ARG1* was highly expressed and strongly induced by LPS, whereas in horse *ARG2* was constitutively expressed at high levels (TPM≈120) but downregulated by LPS stimulation. In each of the ruminant species, *ARG2*, but not *ARG1*, was expressed and strongly induced by LPS. In rats, and all of the large animals, the ornithine generated by arginase activity is likely metabolized further by ornithine amino transferase and ornithine decarboxylase, which are each constitutively expressed at high levels in macrophages. As discussed by Bogdan (5), arginine might also be derived from either the breakdown of peptides by enzymes such as endoplasmic reticulum–associated aminopeptidase 1, carboxy-peptidases M, and D or by resynthesis from citrulline via argininosuccinate synthase 1 (ASS1) and argininosuccinate lyase 1 (ASL). *Ass1*-deficient mice are also deficient in NO production and antimicrobial activity, and this pathway is required to overcome the degradation of arginine by Arg1 (30). This pathway is likely conserved in rats, because *Asl* was constitutive, and *Ass1* was strongly induced by LPS in the rat macrophages. However, in human macrophages, both *ASL* and *ASS1* were on the limits of detection, and *ASS1* was also very low in the other large animals.

In human macrophages, the production of the NOS2 cofactor, THB/BH_4_, is apparently constrained by very low expression of the synthetic enzymes PTS and SPR. Indeed, in the FANTOM5 data, *SPR* expression is very low, and *PTS* was barely detectable in monocytes or macrophages under any conditions. Activation of human macrophages by LPS produced a massive induction of *GCH1*, but previous reports indicate the major product is neopterin rather than THB4 (22, 23). Early studies identified serum and urinary neopterin as a marker of immune activation in human and other primates, where this product was undetectable in rodents (31). More recently, neopterin was detected in the serum of LPS-challenged pigs, whereas there was only a marginal and transient increase in serum NO (32).

*Gch1* was also strongly induced in rat macrophages by LPS, as previously observed in both BMDM and peritoneal macrophages in mice (7); see also data on www.biogps.org/dataset/GSE10246. In the ruminants and horses, *GCH1* was expressed constitutively, but was further induced (~4-fold) only in cattle. In pigs, *PTS* mRNA was detected at very high levels (TPM≈140). *GCH1* was not annotated in the pig genome (version 10.2) when expression profiles of pig and human macrophages were previously compared (12). Unlike human macrophages, pig macrophages expressed low levels of *GCH1* constitutively but it was not induced by LPS.

### Gain and loss of candidate enhancers in the NOS2 promoter

We and others have shown that variation in LPS-inducible gene expression in humans, mice and pigs, including that of *NOS2*, is associated with major differences in promoter architecture including the gain and loss of candidate enhancers (7, 12). The inducible arginine transporter *Slc7a2*, which is essential for NO production in mouse macrophages, provides another example. In the FANTOM5 CAGE data, this gene is highly expressed in liver but undetectable in myeloid cells in any state of activation, whereas in mouse it is expressed at similar levels in liver and activated macrophages. Inducible activity of mouse Slc7a2 varies between mouse strains, associated with alterations in a distal purine-rich promoter element (33). This element is not conserved in the rat promoter, and indeed the promoter regions of mouse, rat, pig, and human have diverged substantially (Supplemental Fig. 2).

**Figure 3:**
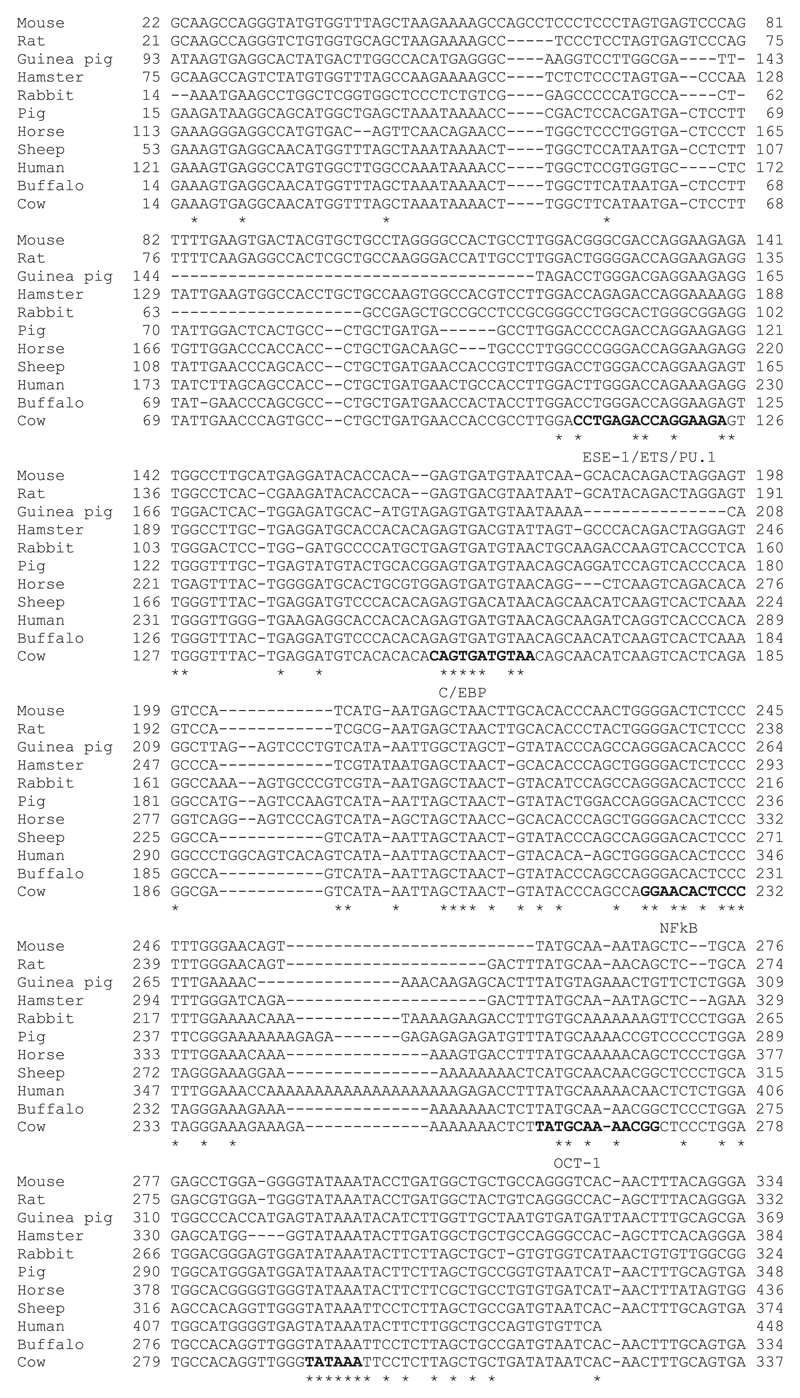
Alignment of the *NOS2* proximal promoter region across species. A 323 bp region of the proximal *NOS2* promoter was aligned between 11 species that show LPS-induced *NOS2* gene expression or not. Transcription factor binding sites, PU.1, C/EBP, NF-κB, and OCT1 and the TATA box are indicated in bold. Asterisks indicate bases conserved across the species.

We were especially interested in the mechanisms distinguishing *NOS2* induction between the four relatively closely related ruminant species. Fig. 3 shows alignment of the proximal promoter regions across species that do, or do not, show induction of *NOS2* mRNA in response to LPS in our experiments or previous studies. In each species there is a TATA box. Despite the relatively low overall conservation, the proximal promoter elements that have been implicated in transcriptional regulation (13, 34), including NF-κB, Oct1, and C/EBP motifs, are conserved and do not correlate with LPS inducibility. Accordingly, it seems likely that differences among species relate to variation in more distal regulatory elements, such as the enhancer located around −1 kb upstream in the mouse genome.

**Figure 4:**
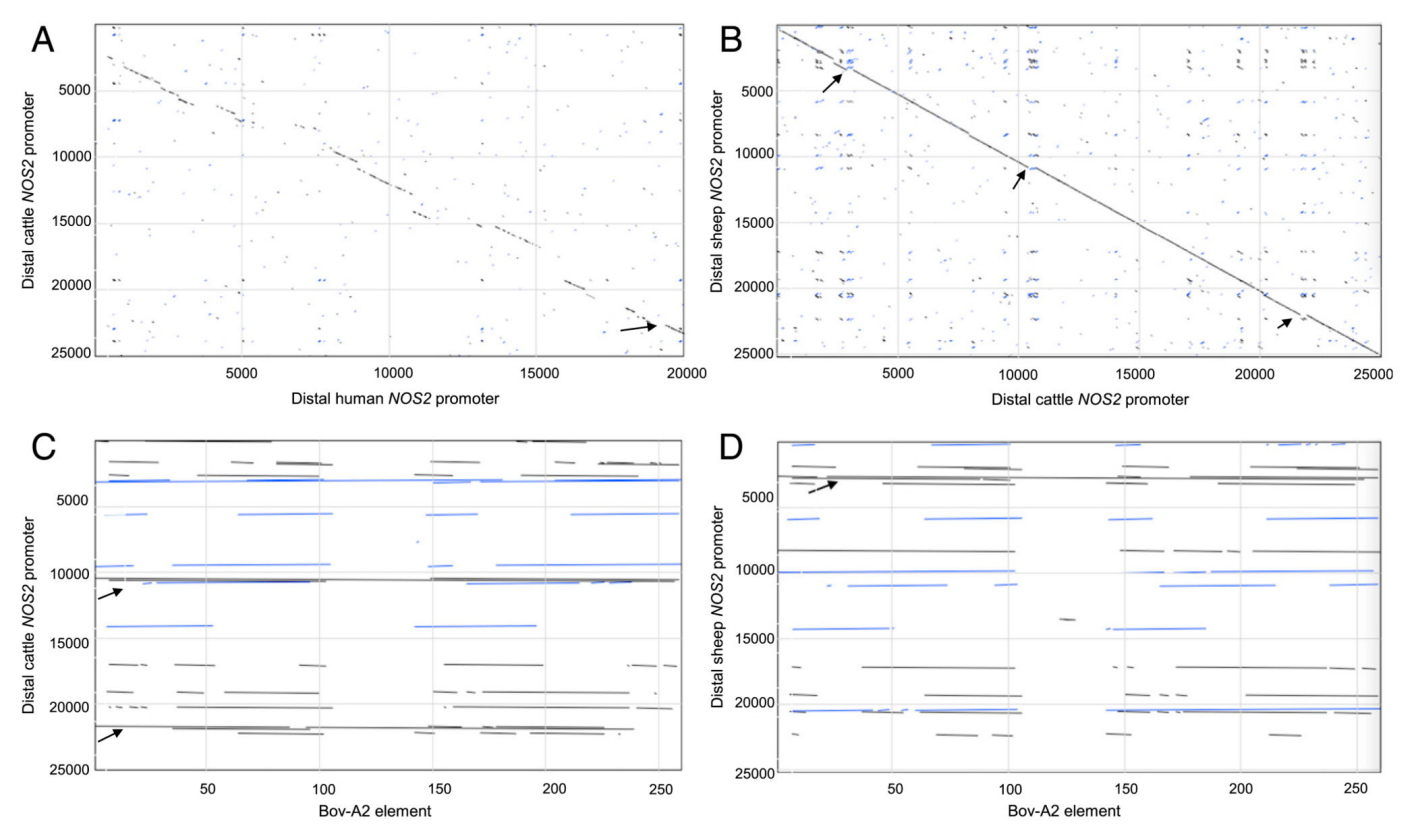
Alignment of distal *NOS2* promoters from cattle, human, and sheep, and the Bov-A2 element. *NOS2* promoter sequences were obtained from Ensembl. (**A** and **B**) show pairwise dot-matrix alignment of 25 kb sequences upstream of *NOS2* transcription start site from (A) cattle (*y*-axis) versus human (*x*-axis), and (B) sheep (*y*-axis) versus cattle (*x*-axis). The arrow in (A) indicates the relative location of the mouse *Nos2* enhancer. In (B), small insertions showing misalignment are indicated by arrows. (**C** and **D**) show the alignment of the Bov-A2 element with the cattle (C) and sheep (D) 25 kb *NOS2* promoter regions. Arrows indicate regions occupied by partial or complete Bov-A2 elements.

Fig. 4A shows a pairwise dot-matrix alignment of distal *NOS2* promoters from cattle and human. An arrow indicates the relative location of the mouse enhancer, which was previously shown to be poorly conserved in humans and lacked the enhancer activity detected in the equivalent mouse sequence (34). In this region, cattle, sheep, and pig genomes are similar to human, with multiple substitutions in the putative mouse LPS responsive element. Regions of relative conservation between the human and bovine *NOS2* 5′ flanking region extending up to 25 kb from the transcription start site are interspersed with regions in which there is no detectable alignment. Fig. 4B shows a similar alignment of cattle and sheep, where there is almost perfect conservation with the exception of a number of small insertions. Both the regions of substantial misalignment between the ruminants and other large animals, and the small additional insertions in cattle relative to sheep (and vice versa), are due to the presence of the Bov-A2 SINE retrotransposon, an ancestral element present at up to 200,000 copies in ruminant genomes (35, 36). Fig. 4C shows the alignment of the Bov-A2 element with the cattle *NOS2* promoter region, and Fig. 4D shows the equivalent alignment with the sheep. It is clear that those regions lacking homology with the human promoter are predominantly occupied by partial or complete Bov-A2 elements.

**Figure 5:**
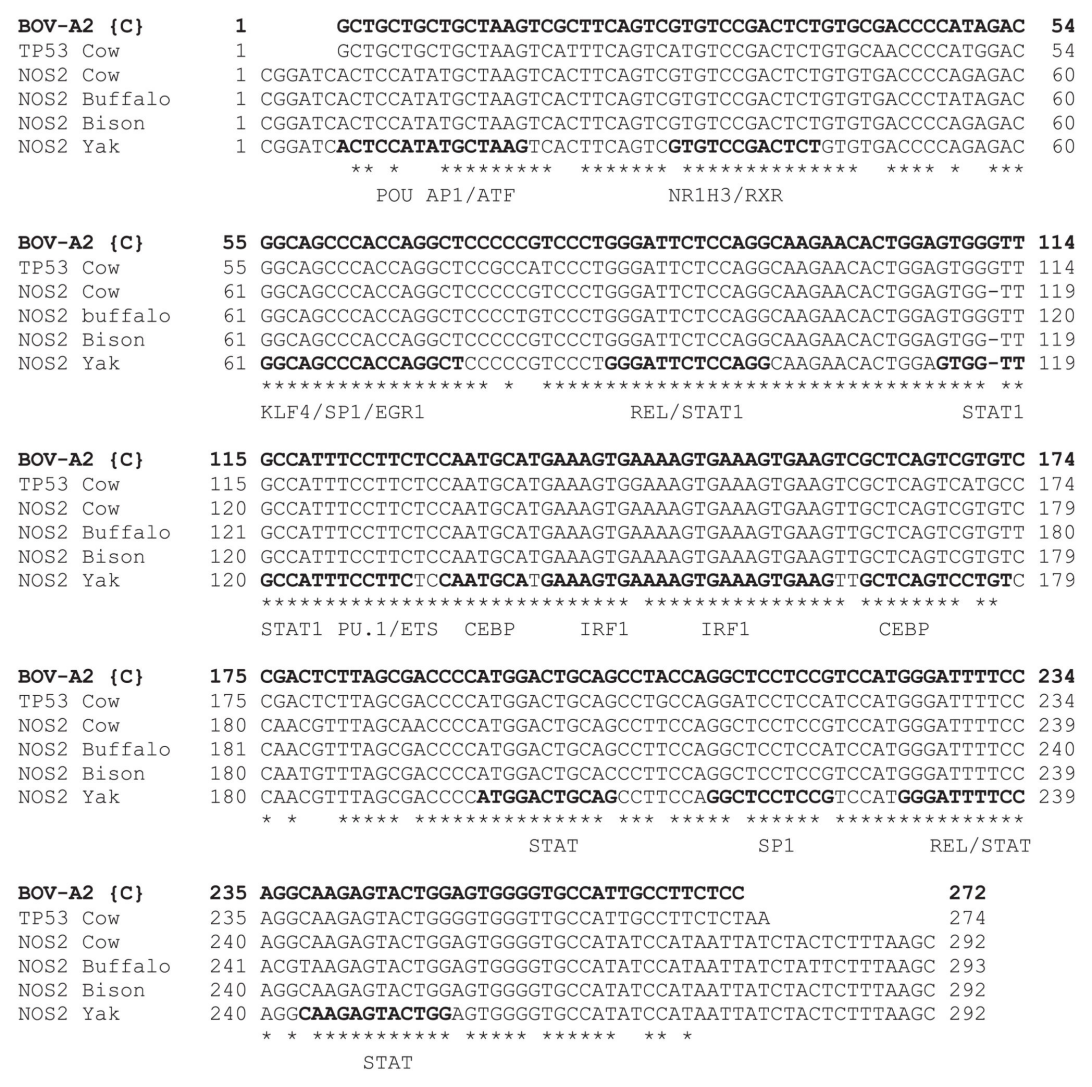
The Bov-A2 element is conserved in the *NOS2* gene of bovid species. A ~300 bp region of the cattle *TP53* gene and *NOS2* gene from cattle, buffalo, bison, and yak were aligned to the consensus BOV-A2 sequence. Candidate transcription factor binding sites derived from analysis with Jaspar are indicated in bold. Asterisks indicate bases conserved across the species.

The absence of a Bov-A2 insertion in the proximal *TP53* promoter region has been implicated in regulated mammary involution and the persistence of lactation in bovids compared with other ruminants through functional STAT1 and NF-κB responsive motifs (37). We have located this proximal insertion in the *NOS2* gene in the bison, water buffalo, and yak genomes, but it was absent in goats. Fig. 5 shows alignments of the *NOS2* Bov-A2 element from four bovid species with the consensus BOV-A2 sequence, and with a distal BOV-A2 sequence extracted from the *TP53* locus. A notable feature is that the direct repeats flanking this insertion are conserved in all bovids, but also in the sheep and goat genomes (data not shown), suggesting that this is a relatively recent insertion whereas the insertion site preexisted in the ancestral ruminant. The aligned sequences in Fig. 5 are also annotated with candidate transcription factor binding sites derived from analysis of the sequence using Jaspar (http://jaspar.genereg.net). Damiani et al. (35) have noted the association of BOV-A2 element insertions with regulatory regions of ruminant genomes and have speculated upon their role in transcription regulation. We reasoned that BOV-A2, containing binding sites for so many macrophage-specific (PU.1, CEBPβ) and inducible (STAT1, IRF1, NF-κB) transcription factors, could contribute to the regulated expression of *NOS2* in ruminants, compared with large (nonruminant) animal species, and that the additional BOV-A2 element located more proximally in the bovine genome could explain the increased expression.

### The bovine NOS2 BOV-A2 element is LPS responsive

**Figure 6:**
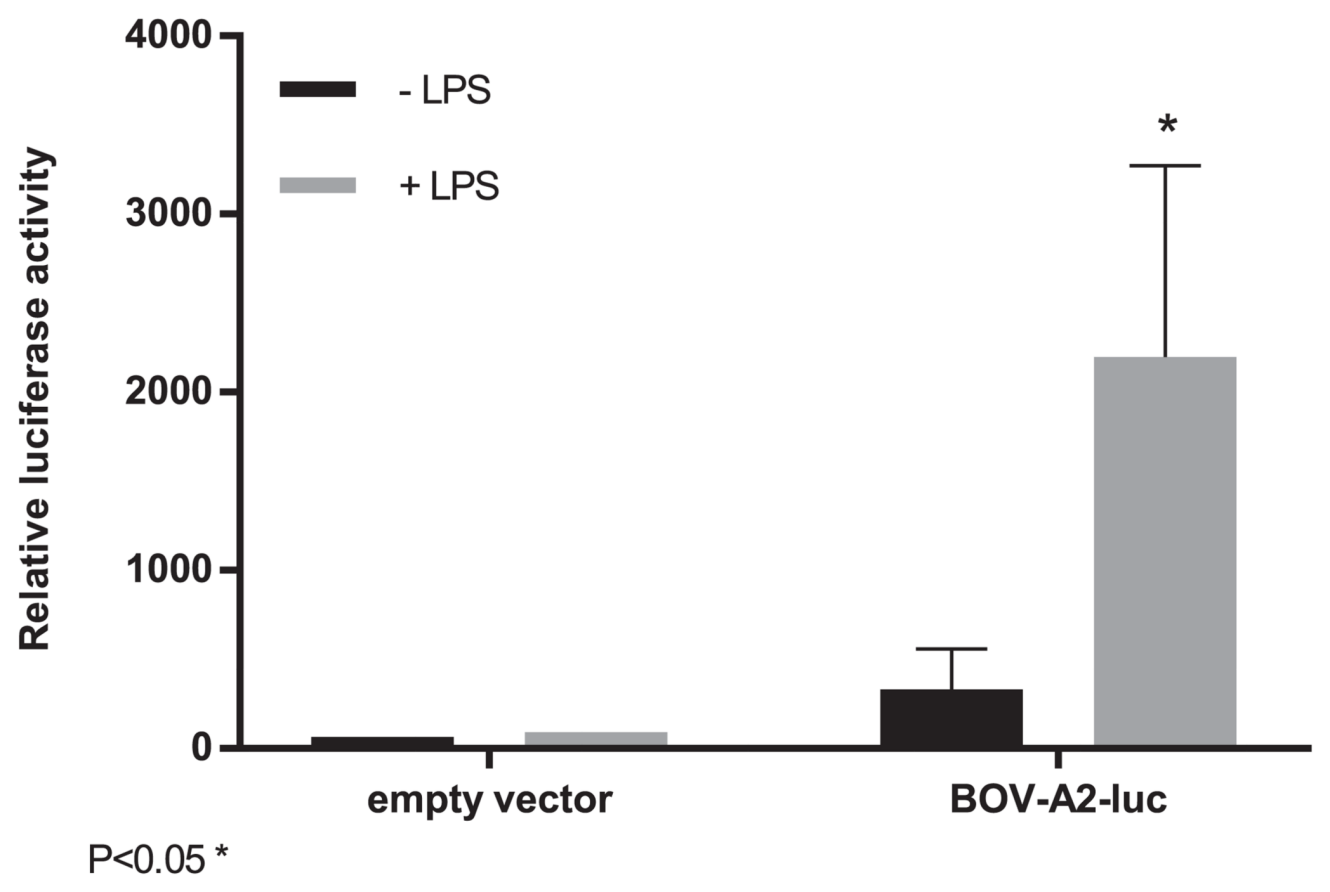
The Bov-A2 element is inducible in response to LPS. RAW 264.7 cells were transfected with a Bov-A2–luciferase enhancer reporter vector or empty vector and stimulated with LPS for 24 h. Relative luciferase activity was measured in control and stimulated cells. The mean relative luciferase activity + SD is shown. This was calculated for replicates and is representative of three independent experiments. Statistically significant difference at 7 h versus 0 h (*t* test; *p < 0.05).

To confirm the activity of the proximal *NOS2* bovine copy of BOV-A2 as a possible regulatory element, we constructed an enhancer/reporter luciferase construct and transfected the LPS-responsive mouse macrophage cell line, RAW264 (38). This line was previously used to demonstrate the lack of activity of the human *NOS2* promoter and enhancer (34). The results of transient transfection analysis are shown in Fig. 6. Compared to the basal promoter, the presence of the candidate BOV-A2 enhancer element produced both constitutive reporter gene activity and increased expression in the presence of LPS. Plasmid DNA can itself induce NF-κB–dependent reporter activity via TLR9 (39), and so the basal activity of the NOS2 Bov-A2 element is most likely partly attributable to activation by this pathway.

## Discussion

We have dissected the transcriptional regulation in macrophages of genes associated with arginine metabolism in a range of species. BMDM from sheep, cattle, water buffalo, goat, horse, pig, and rat were cultured under identical conditions, and stimulated with the same stimulus, LPS. Although it has been suggested that macrophages from different species respond differently to cell culture and that arginine metabolism may be different in vivo, it remains the case that there is large divergence between species and *NOS2* is only one component of the difference. In a recent review of arginine metabolism in myeloid cells (40), the authors discussed the prevalent uptake of arginine by the Y+ amino acid transport system (SLC7A2), the functional importance of inducible arginase 1 (ARG1) in control of arginine availability, the biological importance of NO production in antimicrobial defense and the fact that in macrophages, the NOS2 product, citrulline, can be recycled to arginine via ASS1 and ASL (30). Our analysis of the response of rat macrophages to LPS demonstrates that each of these responses is shared with mice; *Nos2, Arg1, Slc7a2*, and *Asl* were each very highly induced after 7 h exposure to LPS and *Ass1* was constitutively expressed (Table I). However, of the LPS responses observed in rodents, only the induction of *NOS2* and *ARG1* was observed to any extent in any nonrodent species.

Jungi et al. (16) reported previously that bovine macrophages grown from BM or blood monocytes, or isolated from alveolar lavage, were able to induce *NOS2* mRNA and produce NO in response to LPS. In the same study, goat macrophages produced much less *NOS2* mRNA and NO than cattle. We have repeated these studies and extended them to two additional ruminant species, sheep and water buffalo. By contrast to the previous findings, goat macrophages produced detectable NO, despite low expression of *NOS2*, whereas there was no detectable NO production by sheep macrophages (in which *NOS2* was induced to a similar extent) or in horses or pigs, where it was not induced at all. One explanation may be the selective expression of cationic amino acid transporters, *SLC7A1* and *SLC7A2* in goats relative to the other species (Table I).

The comparative analysis we have presented strongly supports the view that the divergent expression of *NOS2* and other genes is a consequence of the evolution of *cis*-acting regulatory elements (7, 12) rather than an idiosyncratic feature of cell culture systems. As shown in Supplemental Fig. 2, the differential regulation of the inducible arginine transporter, *Slc7a2*, in rodent macrophages is associated with the presence of purine-rich binding motifs for the macrophage transcription factor, PU.1, which were shown previously to be functional (31). The unique regulated expression of *ARG2* in ruminant species and horses is also associated with large-scale promoter divergence to the extent that there is little alignment outside −1 kb even between cattle and sheep. There is a BOV-A2 insertion around −3 kb in sheep and goats that is not present in cattle. Multiple insertions of the BOV-A2 retrotransposon produce major differences between the human and pig *NOS2* promoter regions, which are not LPS inducible, and the ruminant *NOS2* promoters, which are. Our data suggest that the recent insertion of a proximal BOV-A2 element in the bovid lineage, shared by cattle, water buffalo, yak, and bison, could contribute to the elevated expression and greater inducibility of *NOS2* in these species. A more global comparative analysis of the RNA-seq datasets may reveal other examples of functional gain and loss of the BOV-A2 element that contribute to species-specific inducible gene expression in ruminant macrophages. The differences in arginine metabolism and production of NO could potentially underlie species-specific susceptibility to pathogens. For example, sheep are considered much more susceptible than cattle to the parasite *Toxoplasma gondii* (41) whereas NO is strongly implicated in both resistance to the parasite, and pathology, in mice based upon analysis of *Nos2* knockouts (42). In overview, our findings extend the evidence that rodents are not always appropriate models for understanding host defense and pathology in other mammalian species including humans (7, 12).

## Supplementary Material

The online version of this article contains supplemental material.

Supplementary Figures and Table

## Figures and Tables

**Table I:** Expression levels of transcripts encoding enzymes associated with arginine metabolism and the production of NO

		RNA-Seq							
		Sheep		Goat		Cattle		Buffalo	
Gene Name	Description	0 h	7 h	0 h	7 h	0 h	7 h	0 h	7 h
ARG1	Arginase 1	0.04	0.05	2.64	2.73	6.05	5.84	0.02	0.01
ARG2	Arginase 2	7.40	56.27	11.82	41.06	48.48	347.99	30.06	153.54
ASL	Argininosuccinate lyase	31.48	14.75	25.40	17.78	38.21	17.99	19.72	6.50
ASS1	Argininosuccinate synthase 1	19.39	10.18	4.38	3.72	10.71	7.65	0.05	0.32
GCH1	GTP cyclohydrolase 1	17.14	32.88	8.31	10.15	22.94	86.98	13.66	10.90
NOS2	NO synthase 2, inducible	0.55	19.09	3.78	19.69	2.14	901.20	3.25	301.37
OAT	Ornithine aminotransferase	362.00	324.62	164.61	167.15	1166.67	854.75	204.40	142.74
ODC1	Ornithine decarboxylase 1	92.12	269.73	340.63	376.01	45.46	18.26	119.02	53.83
PTS	6-pyruvoyltetrahydropterin synthase	17.49	13.66	62.01	62.61	27.53	11.91	36.36	16.32
SLC3A2	Solute carrier family 3, member 2	161.19	167.79	209.91	210.11	98.20	62.87	168.22	146.74
SLC7A1	Solute carrier family 7, member 1	7.91	10.02	38.08	45.94	7.37	6.99	19.95	49.59
SLC7A2	Solute carrier family 7, member 2	0.01	0.04	5.88	15.73	0.04	0.18	0.18	2.03
SLC7A5	Solute carrier family 7, member 5	9.01	21.67	115.47	106.67	5.83	3.46	7.74	7.46
SLC7A7	Solute carrier family 7, member 7	283.90	161.32	109.13	60.78	220.58	120.25	114.72	103.68
SPR	Sepiapterin reductase	21.00	13.80	78.29	124.30	20.47	4.60	37.28	13.15
				RNA-Seq				CAGE	
	
		Horse		Pig		Rat		Human	
	
Gene Name	Description	0 h	7 h	0 h	7 h	0 h	7 h	0 h	7 h

ARG1	Arginase 1	0.10	0.05	70.62	379.11	76.63	1041.60	0.00	0.00
ARG2	Arginase 2	119.23	59.72	13.56	11.91	3.35	1.88	3.10	1.30
ASL	Argininosuccinate lyase	24.06	14.48	13.93	10.35	69.63	50.24	5.80	0.70
ASS1	Argininosuccinate synthase 1	25.85	20.34	0.02	0.04	2.38	250.12	0.00	0.00
GCH1	GTP cyclohydrolase 1	24.77	31.89	9.82	9.67	24.17	189.57	0.70	391.00
NOS2	NO synthase 2, inducible	0.00	0.01	0.66	1.77	59.03	4961.79	0.00	0.00
OAT	Ornithine aminotransferase	103.56	68.81	95.74	83.74	104.93	82.10	83.00	57.00
ODC1	Ornithine decarboxylase 1	98.48	39.73	241.26	195.93	64.55	45.63	5.30	1.30
PTS	6-pyruvoyltetrahydropterin synthase	27.98	16.30	143.49	141.30	11.51	13.01	0.50	0.00
SLC3A2	Solute carrier family 3, member 2	168.35	120.18	238.46	248.84	191.23	289.86	115.00	123.00
SLC7A1	Solute carrier family 7, member 1	13.27	9.72	5.45	4.68	20.02	14.00	0.00	0.00
SLC7A2	Solute carrier family 7, member 2	0.01	0.05	1.00	0.47	46.31	843.81	0.00	0.00
SLC7A5	Solute carrier family 7, member 5	8.97	7.28	13.09	8.81	17.71	14.76	2.70	75.00
SLC7A7	Solute carrier family 7, member 7	57.12	46.60	136.68	198.99	16.97	16.00	71.00	25.00
SPR	Sepiapterin reductase	10.20	3.55	13.08	8.26	10.08	9.95	21.00	1.80

Expression levels are represented as TPM, and are mean values for each condition from multiple animals. Sheep, *n* = 6; goat, *n* = 3; buffalo, *n* = 4; cattle, *n* = 4; horse, *n* = 3; pig, *n* = 3; rat, *n* = 3; human, *n* = 3. Human data were generated by CAGE-seq, as previously described (10), and all other data were generated by RNA-seq as described.

## Abbreviations used in this article

ARG1: arginase 1
ASL: argininosuccinate lyase 1
ASS1: argininosuccinate synthase 1
BM: bone marrow
BMDM: BM-derived macrophage
GCH1: GTP cyclohydrolase 1
PTS: 6-pyruvoyl tetrahydrobiopterin synthase
RNA-seq: RNA sequencing
THB4: tetrahydrobiopterin
TPM: transcript per million
