# Supplementary material for: Species-Specific Transcriptional Regulation of Genes Involved in Nitric Oxide Production and Arginine Metabolism in Macrophages
Source: Immunohorizons. Author manuscript; Available in PMC 2018 Nov 20. (PMC6245571; doi:10.4049/immunohorizons.1700073)
Supplement: Supplementary Figures and Table [file NIHMS80278-supplement-Supplementary_Figures_and_Table.pdf]

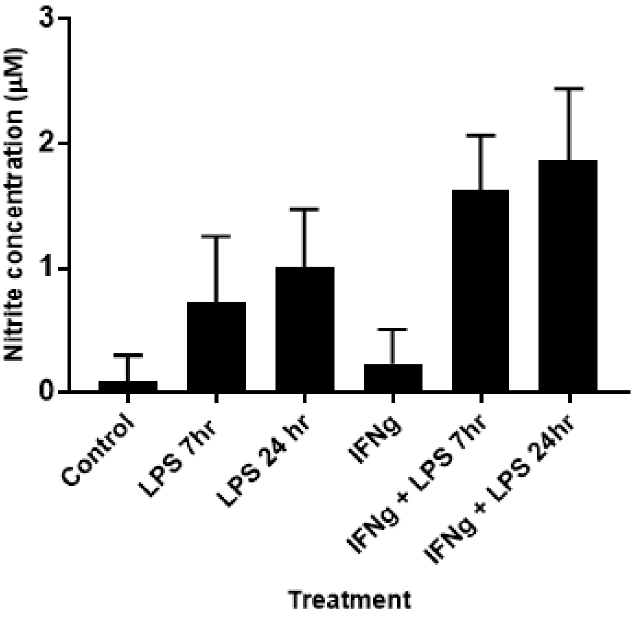


Supplementary Figure 1. IFNγ does not significantly induce NO production in horse BMDM. Horse BMDM were stimulated with LPS at 0, 7, and 24 hr with or without IFNγ treatment. Nitrite concentration was measured by Griess assay. T-test was performed between LPS and LPS + IFNγ treatment at each timepoint, however differences were not statistically significant. Error bars represent the SD of the mean of triplicates.

```
Human SLC7A2      GA---AAATTAACAGGCTTTCACATTGTGACTT-AATCTTATCAGAGACTCCTAAAGTAAACAATAGCGCAGTAGGAGAA
Rat Slc7a2        TAGAAAAATAAA--GAGTTTCACATTGTGACTTTAATCTTATCAGAGACTCCAAAAGCAAGCAATAGCACAGAACGAGAA
Mouse Slc7a2      TA---AAATCAA--GAGTTTCACATTGTGACTTTAATCTTATCAGAGACTTCTAAAGCAA---ATAGCACAGAATGAAAA
Pig SLC7A2        TA---AAATTAACAGAGTTTCATATTGTGTCTTTAATCTTATCAGAGACTCCTAAAGTAAACAATAGCGCAGTATGAGAA
                   *   **** **  *  ***** ****** *** **************** * **** **   ***** *** * ** **

Human SLC7A2      ACCCTGATTGTGTAACTTCTGCTCTTGCTCCCCTGGAAATA----ATGTCATTTCCTA--TAAACCCACCCCCAAACAAT
Rat Slc7a2        AGCTCGATTGTGTAACTTCTGTTTTTGCTGGCTAGGAAATACCAAGTATCCTTTTCTT-AAAAACCCACCTCCAAATAAC
Mouse Slc7a2      ACCTCGATTGTGTAACTTCTGTTTTTGCTGGCTATGAAATACCAAGTATCCGTTTCTTTAAAAACCCACTTCCAAATAAC
Pig SLC7A2        AACTTGGTTGTGTAACTCTTG--------------GAAATAGCAAATGTCGCTTTCTT--ATAGCTCGCCTCTAAGTCAT
                  * *  * **********  **              ******     * **  ** **     * * * *  * **   *

Human SLC7A2      CGTGACTACGTATTCATACCACTGGAGTCTTCCAAAATAGCAACTGCACATTA-------------------TTTATCAA
Rat Slc7a2        CGTGACTGCCTGCCTGCCTATTCACAAGTCT-----GAAGTCCTCGAGTTTTGCTTTCACTAT------GATTTCATTAC
Mouse Slc7a2      CATGACTATG-ACCTGCCTATTCACGAGTCT-----GGAATCTTCAAGTTTTG-----------------ATTTCATTAC
Pig SLC7A2        CAGGTCTACCTGTTCACTTCACTGGGACTTTCCAA-ATAGCAACTAAATATTGCAATAAACGGTTTACAGGTTTTATTGG
                  *  * **                       *       *           **                    ** **

Human SLC7A2      CGTTTAGTTTGCATTT-GACAAAGCACATCCACCTGGGCTTCCATTTATCATTGCTATTATTATATTTTATTATTATTTT
Rat Slc7a2        AGAAAGTGATACTTTT-GACAAAGCACATCTATCTAGGCTTCT--------AGTTTATTATAATCATATACTACTATTTT
Mouse Slc7a2      AGAACGTGATACTTTTTAACAAAGCTCATCTATCTGGGCTTCT--------AGTTTATTATGATCATATACTACTATTTT
Pig SLC7A2        TGAAA-TAATACTATTTGACAAAGCACTCCCATCTGGGTTCTTTTATCACGAGGTCGTTGTTATATTTTACTATTATTTT
                   *       * *  **  ******* *  * * ** ** *                 ** * **  * ** ** ******

Human SLC7A2      CTTATTTCCAAAATCT--TTGTAGAATGGAAAGTAAACGGGGGGAC---------------------CAAAACCCCACTT
Rat Slc7a2        TATGTTCCCAATTCTCAATGAGAGAGT----------------GAGGGAAAGAGGGAGGGAGAGGAACAAAATCTTGCTT
Mouse Slc7a2      TATGTTCCCAATTCTCAATGAGAGAGGGAGAGAAAGAGGGAGGGAGGGAGAGAGGGAAGAAGAGGAAAAGAATCCTGCTT
Pig SLC7A2        CTTATTTACAAATTCC--TCGCAGACTAAAACCTAAATCAAGTCAG------------------GGACAAAATCCCACCT
                    * **  ***       *   ***         * *    *  *                       * ** *   * *

Human SLC7A2      A---GAACTCTGCTTAGAAGATTCGGAAATGCCCTTG---------------AGGTTTGGTCCTCCCAGGAGAGCACGAA
Rat Slc7a2        AGAAGAAACCT-CAGAAATGCCCTGCCCCCACCCCCCCACACACACACATTGAGATTGGGTTCTGCCAAGAGA-----GC
Mouse Slc7a2      AGAAGAAACCT-CAGAAATGTCCCCCACCCCACCCCC---------------AGATTGGGTTCTGCCAAGGAAAAGGGGC
Pig SLC7A2        GAAGGAACTCTGCTTAGAAGCCTCGGAGATGGCCCTG---------------AGTTTGGGTCCT-CCATGGAAGGCAAGA
                         ***  ** *  * * *            **                  ** ** *** ** *** *  *

Human SLC7A2      GTTTATCCTCGCGCGCAG-----CCTCTCTTCCCCGGCCCGCGCCCACCG-----------------GCCTAGCCCGGGG
Rat Slc7a2        GTTTTCCCTCTGCCAAG------CCCTGCGTCTCGGACCCACGCCCACCC---------ACCTACCCACCCAGC---GAG
Mouse Slc7a2      ATTTCCCCTCTGCCAAA------CCCTGCGTCTCCGACCCACGCCCACCC---------ACC-------CCAGC---GGG
Pig SLC7A2        ATTCACCCTCCGCTGGGTGTGCCCTCCTCCCGCCCGACCTGCCCCCACTGCTCTCCGGAGCCCACCCGCCCAGCCCCAGG
                   **   ****             *    *    * * **  * *****                     * ***     *

Human SLC7A2      CTAGCGCCCGC-CCACGTGTGCTCGGCTCCAGGCA--AACCCCG---------CTGAGCAGCGGCCGCACACCGCCCACC
Rat Slc7a2        CAAGCTTGTAA-CCACGTGTGCCTACACCCTGGCCCTGACCCTA------CAGAGGCGCGGAG-CTAGACACCGCCTTCC
Mouse Slc7a2      CAAGCGTGTAATCCACGTGCGCGCGCGCCCTGATCCTGACCCTGACCTTTCAGAAGCTTGGAG-TTAGACACCGCCTTCC
Pig SLC7A2        CAAGCGCACGC-CCACGTGTGCCACCGCTCGGGGAGGGAGACTGAAC---TAGCGGCGTTGCGACCGGACGCCGCCTACC
                  * ***       ******* **       * *      *  *             *    * *     ** *****  **

Human SLC7A2      CCGGGGATTGGTCAGCGCGGCCG--GGGCCCGGCGGGAGGCGGGCTC-GGGGTCGCGTTCCGGGAGCGCGGAGGAGGCGG
Rat Slc7a2        T-GGAATCT---CAGCGTGCCTG--------GAGGGGACGAGCGCTCCAGGAGGGGGTCGCGAGCGCGAGGGAGGGGCGG
Mouse Slc7a2      T-GGAGTCT---CTGCGCGCCCGCCCGCCGAGCCGTGACAAACGCTCCAGGCGAGGGTCGCGAGCACGAGGGAGGGGCGG
Pig SLC7A2        C-CAGGATTGGCCGGCGCGGTGG--------GGCGGGAGGCGGGCTCGGGTGGGGGGGTCTGAGAGCGCGGGAGAGAGAA
                          *   * *** *   *        *  * **     ****  *    * *    * *  ** **  * *

Human SLC7A2      TGCCGCCC---------------GGCCCCGC-GCCCCGCCC---------CCGCCCCGG-GTGGCTACACAGAGGG----
Rat Slc7a2        AACGGCCCTGCCCTCGAGGCCCCGCCCTCGCGGCCCCGCCC---TTCGCCCCGCCCCTCCGCTTCTACTCAGAGGGTTGC
Mouse Slc7a2      GACGGCCC------------CCGCCCCTCGCTGCCCCGCCCCACTCCGCCCCGCCCCTCCGCCTCTACTCAGAGGG----
Pig SLC7A2        GGGGGGCG----------GTTCCACCCGGGCAGCCCCGCCC--------CCGGCGCGGCTACACCAGCT-----------
                      * *                  **  ** *********         * ** *        *  *

Human SLC7A2      ----GCGCCCACGTGCCCAGCCC
Rat Slc7a2        CCACGTGCCCACGTGCCCAGCCC
Mouse Slc7a2      ----GTGCCCACGTGCCCAGCCC
Pig SLC7A2        ----GCGCCCACGTGCCCAGTCC
                      * ************** **
```

Supplementary Figure 2. Alignment of proximal promoters of *SLC7A2* in multiple species. Promoter sequences were downloaded from ENSEMBL. The last base corresponds to the peak of transcription initiation for the gene in mouse and human based upon the data from the FANTOM Consortium (Ref 10) on the Zenbu browser (http://fantom.gsc.riken.jp/zenbu/. ). Red highlight indicates the purine-rich element highlighted in Ref 31, which is not conserved in rat. However, note that both species have at least two candidate PU.1 sites containing the GGAA core (underlined).

Supplementary Table 1. Source of reference transcripts for RNA-seq processing.

| Species | Genome version | Source | URLs | No. of transcripts used as input for the 'first pass' Kallisto index | No. of transcripts not detectably expressed (TPM > 1) in any library after the first round of quantification with Kallisto * | After the second round of quantification with Kallisto | | | |
|---|---|---|---|---|---|---|---|---|---|
| | | | | | | Avg. no. of protein-coding genes expressed (TPM > 1) in BMDMs 0 hr post-LPS | % of protein-coding genes expressed (TPM > 1) in BMDMs 0 hr post-LPS | Avg. no. of protein-coding genes expressed (TPM > 1) in BMDMs 7 hr post-LPS | % of protein-coding genes expressed (TPM > 1) in BMDMs 7 hr post-LPS |
| Cattle (*Bos taurus*) | UMD 3.1 | Ensembl (version 83) | [1] protein-coding, pseudogene and processed pseudogene transcripts downloaded from: ftp://ftp.ensembl.org/pub/release-83/fasta/bos_taurus/cdna/Bos_taurus.UMD3.1.cdna.all.fa.gz, [2] non-protein coding transcripts obtained from Ensembl BioMart, filtered by type: lincRNA, miRNA, misc_RNA, Mt_rRNA, Mt_tRNA, rRNA, snoRNA, snRNA | 26,740 | 5,050 | 11,940 | 59.72 | 11,276 | 56.4 |
| Goat (*Capra hircus*) | ARS1 | NCBI | ftp://ftp.ncbi.nlm.nih.gov/genomes/all/GCF/001/704/415/GCF_001704415.1_ARS1/GCF_001704415.1_ARS1_rna.fna.gz | 47,193 | 964 | 11,875 | 62.96 | 11,662 | 61.83 |
| Horse (*Equus caballus*) | EquCab 2.0 | Ensembl (version 85) | [1] protein-coding, pseudogene and processed pseudogene transcripts downloaded from: ftp://ftp.ensembl.org/pub/release-85/fasta/equus_caballus/cdna/Equus_caballus.EquCab2.cdna.all.fa.gz, [2] non-protein coding transcripts obtained from Ensembl BioMart, filtered by type: lincRNA, miRNA, misc_RNA, Mt_rRNA, Mt_tRNA, rRNA, snoRNA, snRNA | 29,196 | 4,350 | 11,622 | 56.83 | 11,304 | 55.28 |
| Pig (*Sus scrofa*) | Sscrofa 11.1 | Ensembl (version 90) | [1] protein-coding, pseudogene and processed pseudogene transcripts downloaded from: ftp://ftp.ensembl.org/pub/release-90/fasta/sus_scrofa/cdna/Sus_scrofa.Sscrofa11.1.cdna.all.fa.gz, [2] non-protein coding transcripts obtained from Ensembl BioMart, filtered by type: IG_C_gene, IG_V_gene, lincRNA, miRNA, misc_RNA, Mt_rRNA, Mt_tRNA, ribozyme, rRNA, scaRNA, snoRNA, snRNA, sRNA, TR_J_gene, TR_V_gene | 49,436 | 8,881 | 12,732 | 56.99 | 12,316 | 55.12 |
| Rat (*Rattus norvegicus*) | Rnor 6.0 | Ensembl (version 88) | [1] protein-coding, pseudogene and processed pseudogene transcripts downloaded from: ftp://ftp.ensembl.org/pub/release-88/fasta/rattus_norvegicus/cdna/Rattus_norvegicus.Rnor_6.0.cdna.all.fa.gz, [2] non-protein coding transcripts downloaded from ftp://ftp.ensembl.org/pub/release-88/fasta/rattus_norvegicus/cdna/Rattus_norvegicus.Rnor_6.0.cdna.all.fa.gz | 47,106 | 13,162 | 11,843 | 53.23 | 11,568 | 51.99 |
| Sheep (*Ovis aries*) | Oar 3.1 | Ensembl | [1] protein-coding, pseudogene and processed pseudogene transcripts downloaded from: ftp://ftp.ensembl.org/pub/release-83/fasta/ovis_aries/cdna/Ovis_aries.Oar_v3.1.cdna.all.fa.gz, [2] non-protein coding transcripts obtained from Ensembl BioMart, filtered by type: lincRNA, miRNA, misc_RNA, Mt_rRNA, Mt_tRNA, rRNA, snoRNA, snRNA | 29,118 | 193 | 12,409 | 59.31 | 12,222 | 58.42 |
| Water buffalo (*Bubalus bubalis*) | UMD CASPUR WB 2.0 | NCBI | ftp://ftp.ncbi.nlm.nih.gov/genomes/all/GCF_000471725.1_UMD_CASPUR_WB_2.0/GCF_000471725.1_UMD_CASPUR_WB_2.0_rna.fna.gz | 45,502 | 99 | 10,659 | 53.17 | 9,879 | 49.28 |

* note that BMDM data for the sheep, water buffalo and goat are derived from large-scale expression atlases for each species, each comprising hundreds (goat) or thousands (sheep, water buffalo) of libraries spanning multiple tissues and cell types. For these species, therefore, there are few transcripts undetected in every library of their respective atlas.
